# Monocyte Subsets: Phenotypes and Function in Tuberculosis Infection

**DOI:** 10.3389/fimmu.2018.01726

**Published:** 2018-07-30

**Authors:** Pavithra Sampath, Kadar Moideen, Uma Devi Ranganathan, Ramalingam Bethunaickan

**Affiliations:** ^1^Department of Immunology, National Institute for Research in Tuberculosis, Chennai, India; ^2^International Center of Excellence in Research, National Institute for Research in Tuberculosis, National Institutes for Health, Chennai, India

**Keywords:** monocyte subsets, CD14^+^ monocytes, CD16^+^ monocytes, biomarkers for tuberculosis, monocyte to lymphocyte ratio, monocyte signatures, *Mycobacterium tuberculosis*

## Abstract

Monocytes are critical defense components that play an important role in the primary innate immune response. The heterogeneous nature of monocytes and their ability to differentiate into either monocyte-derived macrophages or monocyte-derived dendritic cells allows them to serve as a bridge between the innate and adaptive immune responses. Current studies of monocytes based on immunofluorescence, single-cell RNA sequencing and whole mass spectrometry finger printing reveals different classification systems for monocyte subsets. In humans, three circulating monocyte subsets are classified based on relative expression levels of CD14 and CD16 surface proteins, namely classical, intermediate and non-classical subsets. Transcriptomic analyses of these subsets help to define their distinct functional properties. Tuberculosis (TB) is a disease instigated by the deadly pathogen *Mycobacterium tuberculosis*. Current research on monocytes in TB has indicated that there are alterations in the frequency of intermediate and non-classical subsets suggesting their impact in bacterial persistence. In this review, we will focus on these monocyte subsets, including their classification, frequency distribution, cytokine profiles, role as a biomarker and will comment on future directions for understanding the salient phenotypic and functional properties relevant to TB pathogenesis.

## Introduction

Mononuclear cells (monocytes/macrophages) are professional phagocytes that are highly skilled in defense against many pathogens including *Mycobacterium tuberculosis* (MTB). In MTB infection, the phenomenon of granuloma formation helps to isolate organisms and prevent their dissemination, besides, providing a niche for the survival of MTB without damage over long periods of time. Monocytes also participate in many other processes such as homeostasis, tumor surveillance, tissue repair, microbial resistance, maintenance of tissue integrity, apoptosis, necrosis, autophagy, etc. Advances in subset classification of mononuclear cells, their phenotypic and functional properties, and their modulation during disease conditions has stimulated research on identifying monocyte-derived biomarkers for diagnostic and treatment purposes.

## Circulating Monocytes and their Subsets

Human monocytes are bone marrow-derived leukocytes that circulate in the blood and can differentiate into monocyte-derived macrophages and monocyte-derived dendritic cells that govern innate and adaptive immune responses (1). These cells are heterogeneous in nature and exhibit high plasticity. Subset identification of monocytes is based on the relative expression of CD14 [co-receptor for toll-like receptor 4 (TLR4) and mediates lipopolysaccharide (LPS) signaling] and CD16 (Fc gamma receptor IIIa). Flow cytometric phenotyping has identified three different populations of monocytes namely, classical (CD14^++^, CD16^−^), intermediate (CD14^+^, CD16^+^), and non-classical (CD14^+^, CD16^++^) monocytes (2). The three monocyte subsets are phenotypically and functionally different. Earlier studies carried out by Murdoch et al. (3) and Venneri et al. (4) clearly identified two distinct populations of CD16^+^ (intermediate and non-classical) monocytes based on the surface expression and function of the Tie-2 marker. In addition, expression of Slan (6-sulfo LacNac) further distinguished the non-classical and intermediate monocyte subsets (5). However, the function of intermediate population is still not defined with some reports suggesting that they are related to the classical subset and others suggesting that they are related to the non-classical subset. Classical monocytes comprise about 80–95% of circulating monocytes. These cells are highly phagocytic and are known to be important scavenger cells. Intermediate monocytes comprise about 2–8% of circulating monocytes. Their functions include production of reactive oxygen species (ROS), antigen presentation, participating in the proliferation and stimulation of T cells, inflammatory responses, and angiogenesis. Non-classical monocytes comprise about 2–11% of circulating monocytes. They are mobile in nature and patrol the endothelium in search of injury. They can have pro-inflammatory behavior and secrete inflammatory cytokines in response to infection. These cells are also involved in antigen presentation and T cell stimulation (6, 7). Phenotypic and functional differences of these subsets are listed in Table 1.

**Table 1:** Phenotypic and functional differences of classical (CD14^++^, CD16^−^), intermediate (CD14^++^, CD16^+^), and non-classical monocyte (CD14^+^, CD16^++^) subsets.

		Classical	Intermediate	Non-classical	Reference
1	Approximate proportions of total monocytes	80–95%	2–11%	2–8%	Chimen et al. (7)
					Yang et al. (8)
					Wong et al. (9)
					Shantsila et al. (10)
					Ziegler-Heitbrock (11)
					Passlick et al. (12)

2	Size	18 µm	Intermediate	14 µm	Anbazhagan et al. (13)

3	Differentiation potential	Few macrophages and mostly dendritic cells	Non-homogenous population having the potential to differentiate into some macrophages and occasional cells with dendrites	Mostly macrophages	Villani et al. (14)
					Boyette et al. (15)

4	Surface markers and gene signatures	CD14^++^ CD16^−^	CD14^++^ CD16^+^	CD14^+^ CD16^++^	
	ACE	+	++	+	Boyette et al. (15)
	CCR1	+++	+	+/−	Ziegler-Heitbrock (16)
	CCR2	+++	+/−	−	Stansfield and Ingram (17)
	CCR5	++	+++	+/−	Anbazhagan et al. (13)
	CD1d	+++	++	+/−	Hijdra et al. (18)
	CD9	+++	++	+/−	Wong et al. (9)
	CD11a	+	+	++	Tallone et al. (19)
	CD11b	+++	+++	+/−	Wong et al. (6)
	CD11c	+/−	+	+++	Zawada et al. (20)
	CD32	+	+++	++	Rogacev et al. (21)
	CD33	+++	++	+/−	Ulrich et al. (22)
	CD36	+++	++	+/−	Ulrich et al. (22)
	CD40	+/−	+++	++	Ingersoll et al. (23)
	CD43	−	+	++	Sunderkotter et al. (24)
	CD47	++	+++	++	Ancuta et al. (25)
	CD54	+	+++	++	
	CD62L	+	++	−	
	CD64	+++	+++	+/−	
	CD80	+	+++	+/−	
	CD86	+	+++	++	
	CD97	+	++	+++	
	CD99	+++	+	+/−	
	CD115	+	++	+++	
	CD123	+/−	++	+++	
	CD163	++	+++	+/−	
	CD294	+/−	++	+++	
	CLEC4D	+++	+/−	−	
	CLEC5A	+++	+/−	−	
	CLEC10a	+/−	++	+/−	
	CXCR1	+++	+/−	−	
	CXCR2	+++	+/−	−	
	CXCR4	+++	++	+/−	
	CX3CR1	+/−	++	+++	
	GFRα2	+/−	+++	+	
	HLA-ABC	++	+++	+	
	HLA-DR	+/−	+++	++	
	IL13Rα1	+++	+/−	−	
	MHCII	+	++	+	
	PSGL-1	++	+	+	
	P2RX1	+/−	++	+++	
	Siglec10	+/−	+	+++	
	SIRPα	+	++	+++	
	SLAN	+	+/−	+++	
	Tie2	−	+	+/−	
	TNFR1	+	+++	+	
	TNFR2	+	++	+++	

5	Preferred response to LPS	IL-10, G-CSF, CCL2, CCL5, IL-6, IL-8, TNF-alpha	IL-6, IL-8, TNF-alpha	TNF-alpha, IL-1beta, IL-6, IL-8, CCL3 (in response to virus rather than LPS)	Boyette et al. (15)
					Wong et al. (9)
					Cros et al. (26)

6	Gene signature derived functions	Superior phagocytosis, high MPO activity, high antibody-dependent cell-mediated cytotoxicity, increased skin homing potential, wound healing, coagulation, tissue repair, pro-inflammatory (S-100 proteins), scavenger receptors, C-type lectin receptors, anti-apoptosis, proliferative, response to various stimuli, antibacterial, more plasticity	T cell proliferation and stimulation, superior ROS production, angiogenesis (Tie-2 subpopulation), MHC class II presentation and processing, cell differentiation	T cell proliferation and stimulation (SLAN subpopulation), patrolling behavior *in vivo*, better in Fc receptor-mediated phagocytosis, cytoskeletal rearrangement, complement components, pro-apoptosis, anti-proliferative, negative regulation of transcription, antiviral response, pro-angiogenic behavior	Lastrucci et al. (27)
					Anbazhagan et al. (13)
					Wong et al. (9)
					Wong et al. (6)
					Robbins and Swirski (28)
					Cros et al. (26)
					Martinez (29)
					Mobley et al. (30)
			Present tetanus toxoid and other particulate antigens, produce reactive nitrogen intermediates, increased gut homing potential, increased expression of pro-inflammatory genes and co-stimulatory molecules		

*IL, interleukins; TNF, tumor necrosis factor; G-CSF, granulocyte colony-stimulating factor; CCL, C-C motif chemokine ligand; MPO, myeloperoxidase; CXCR1, C-X-C motif chemokine receptor 1; CXCR2, C-X-C motif chemokine receptor 2; LPS, lipopolysaccharide; ROS, reactive oxygen species*.

Subsets of monocytes have also been characterized and classified by various functional studies. Smedman et al. classified monocyte subsets into two groups based on cytokine induction by lipoteichoic acid and LPS; one larger population of cells secreting interleukin (IL)-1beta, IL-6, TNF-alpha, and CCL4 and a second smaller population of cells secreting GM-CSF, IL-10, and IL-12 p40 (31). Gren et al. using single-cell PCR gene expression identified 22 genes that are expressed by the classical population, 8 that characterize the intermediate population, and 6 that distinguish the non-classical population (32). Resting and activated monocyte subsets can be effectively distinguished using whole cell mass spectrometry fingerprinting (33). All these studies provide a platform to study further key details about monocyte phenotype and function, their behavior during disease and their interactions with pathogens.

## Functional Role of Monocyte Subsets Defined by Transcriptomics and Proteomics

Transcriptomic, and proteomic studies can help to decode the functional variation within monocyte subsets. Classical monocytes mainly promote antimicrobial activity by characteristic upregulation of myeloperoxidase (MPO), lysozyme C precursor (LYZ), S100 calcium binding protein A9 (S100A9), eosinophil cationic protein precursor (RNase3), phospholipase B domain containing 1 (PLBD1), and Cathepsin G (CTSG), at both mRNA and protein levels (13, 34). Expression of pro-inflammatory mediators, particularly, S100A12, S100A9, and S100A8 is a hallmark of this subset, but other stimuli can potentially mediate even tissue repair functions, such as wound healing, angiogenesis, and coagulation (6, 13). Non-classical monocytes exhibit upregulation in the mRNA levels of heme oxygenase 1 (HMOX1), Villin 2 (VIL2), and Src family kinases constituting hemopoietic cell kinase (HCK) and tyrosine-protein kinase Lyn (LYN) and protein levels of actin related proteins (ARP2 and ARP3), HCK and LYN. These proteins phosphorylate the immunoreceptor tyrosine-based activation motif (ITAM) of Fc receptors leading to recruitment of downstream genes necessary for cytoskeletal remodeling suggesting a role for this macrophage subset in Fc receptor-mediated phagocytosis (34). In addition, upregulation of Rho GTPases, RhoC, and RhoF with their activators; guanine nucleotide exchange factors (VAV2 and ARHGEF18) and downstream effectors such as phosphatidylinositol-5-phosphate 4-kinase, type II, alpha (PIP5K2A) and protein kinase N1 (PKN1) suggests that they undergo cytoskeletal rearrangement (6, 13). Non-classical subset do not secrete ROS or cytokines in response to cell-surface toll-like receptors. However, they secrete TNF-alpha, IL-1beta, and CCL3 in response to virus and immune complex containing nucleic acids *via* the MyD88–MEK pathway (26). In depth proteome analysis further supports the established functions of classical and non-classical monocyte subsets (35).

A recent study performed by Villani et al. (14) defines the heterogeneity of intermediate monocyte subset based on single-cell RNA sequencing. They identified four monocyte subpopulations namely, Mono 1 (representing mostly the classical monocytes and some intermediate monocytes), Mono 2 (containing a major proportion of non-classical monocytes together with some intermediate monocytes), Mono 3, and Mono 4. These two newly identified Mono 3 and Mono 4 populations represent a major proportion of intermediate monocyte subsets and have unique expression of a set of genes along with co-expression of Mono 1 markers. Mono 3 subset expresses a unique combination of genes that affect cell cycle, differentiation, and trafficking, including MAX dimerization protein 1 (MXD1), C-X-C motif chemokine receptor 1 (CXCR1), C-X-C motif chemokine receptor 2 (CXCR2), and vascular non-inflammatory molecule 2 (VNN2). Mono 4 subset expresses a cytotoxic gene signature resembling that of natural killer dendritic cells including perforin 1, granulysin, and cathepsin W. Thus, it is evident that the earlier identified intermediate monocyte subset is highly heterogeneous in nature.

## Monocytes in Tuberculosis (TB)

Current research focusing on monocytes and their subsets in TB has found that CD16^+^ monocytes are expanded in TB infection (36). Perturbation of this subset defines the severity of TB. Expansion of CD16^+^ monocytes is reversed with anti-TB treatment (37) suggesting this expansion is caused by microbial or host components (36). By contrast, tuberculin skin test (TST) positive individuals express higher CD14^+^ CD16^+^ monocyte subset than either active TB patients or healthy TST negative controls, suggesting that these cells constitute an innate protective mechanism against TB in such individuals (38). This finding has, however, not been well-reproduced. For example Castano et al. (36), did not find significant differences in the monocyte subpopulations between TST-positive individuals and TB patients except for higher CD11b and low HLA-DR surface marker expression in non-classical monocytes.

Castano et al. (36) focused on the differences among the three monocyte subsets between TB patients and healthy individuals. In their study, the percentage of intermediate and non-classical monocytes was increased and classical monocyte was decreased in TB patients. There was also an alteration in the profile of monocyte subsets in TB patients. Classical monocytes and intermediate monocytes of TB patients exhibited a lower expression of CD11b and CCR5 and higher expression of CD80, CD86, non-specific esterase (NSE), and CCR2 when compared with healthy individuals. In addition, intermediate monocytes showed higher expression of CD40 and CD68. CD16^+^ monocytes did not differentiate into macrophages due to limited expression of maturation and differentiation markers such as CD11b, CD11c, CD33, and CD36 (36). Non-classical monocytes of TB patients exhibited a lower expression of CD11c, CD33, CD36, HLA-DR, and CCR5 and higher expression of CD11b, CD40, CD80, NSE, and CCR2. These studies suggest a potential role of intermediate monocytes in T cell activation, proliferation, and antigen presentation.

When compared to CD16^+^ monocytes, CD16^−^ (classical) monocytes confer anti-mycobacterial immune responses during TB infection such as enhanced *in vitro* migration in response to mycobacterial derivatives, higher production of ROS, higher lung migration index, and induction of strong pulmonary infiltration. It has been suggested that immediate infiltration of these subsets to the infection site and the production of ROS results in reduction of bacterial growth. By contrast, CD16^+^ monocytes are involved in promoting bacterial resilience (39). This subset induces a minimal level of respiratory burst and is unresponsive at early stages of infection due to the lack of chemokine receptors (CCR2) necessary for the migration of this subset to the infection site. Similarly, earlier studies have reported that CD16^+^ subset upregulates CCR2 expression during disease severity which aims to improve their migration ability toward the infection (40). Upregulation of CD11b within CD16^+^ monocytes suggests a possible way for intracellular survival of MTB and the loss of HLA-DR confers inefficient antigen presentation potency which leads to disease severity (41). Differential induction of a response to purified protein derivative (PPD) and MTB has been displayed by monocytes obtained from PPD skin test-positive individuals and active TB patients. PPD could induce apoptosis in both groups of subjects, whereas induction with MTB resulted in necrosis only among active TB patients, suggesting that apoptosis can influence the protective ability of the host (42).

Studies carried out by Balboa et al. (43), revealed that CD16^+^ monocytes from active TB patients have a poor capability to differentiate into functional dendritic cells due to high level of phosphorylated p38 MAP kinase. They have also reported differences in the dendritic cell profile among healthy (DC SIGN^high^ and CD1a^+^) and active TB (DC SIGN^low^ and CD86^high^) subjects. This impairs their differentiation of monocytes to monocyte-derived dendritic cells and their antigen-presenting cell functions (44) resulting in a decrease in their ability to mount strong adaptive responses toward infection.

*Mycobacterium tuberculosis* has the ability to modulate the macrophage response and induce the secretion of anti-inflammatory cytokines such as IL-10 thereby modulating the differentiation of CD14^+^ CD16^−^ monocytes toward the M2 activation program (CD16^+^CD163^+^MerTK^+^pSTAT3^+^) in a STAT3-dependent manner. This leads to enhanced protease-dependent motility making them permissive for intracellular MTB survival with reduced ability to produce pro-inflammatory cytokines (27).

## Cytokine Profile of Monocytes During TB Infection

Only limited studies have focused on the cytokine expression of monocytes and their subsets during TB infection. Upon MTB infection, CD16^−^ monocytes produce IL-10 which results in a higher frequency of CD16^+^ monocytes. Adoptive transfer studies of CD16^+^ and CD16^−^ monocytes within SCID/Beige mice infected with MTB showed that mice receiving CD16^−^ monocytes produce higher IL-10 and TGF-beta and mice receiving CD16^+^ monocytes produced higher TNF-alpha. CD16^−^ monocytes exhibit both pro- and anti-inflammatory cytokine production besides leukocyte recruitment under infectious conditions, whereas, CD16^+^ monocytes induce IL-1beta production and leukocyte recruitment under non-infectious conditions (39). In work by Castano et al. (36), classical monocytes produce less TNF-alpha and more IL-10 whereas intermediate and non-classical monocytes produce less IL-10 and more TNF-alpha. CD16^+^ monocytes are involved in promoting the disease, but *in vitro* production of TNF-alpha by these cells helps to control TB infection. In addition, differential cytokine production by CD16^+^ monocytes was observed in response to live vs. dead MTB. Dead MTB induced lower TNF-alpha and IL-8 compared to live MTB as a result of differences in their cell wall structure and components (45). However, it is still not known which subset of CD16^+^ monocytes is actually producing TNF-alpha.

## Monocyte to Lymphocyte Ratio (ML Ratio)

Monocyte to lymphocyte ratio is considered an important criterion to determine the immune efficiency of an individual during infectious conditions and is easily quantified in the peripheral blood. During MTB infection, there have been evolving reports suggesting that an increased ratio of monocytes to lymphocytes in comparison with healthy donors denotes the severity of active TB. A high ML ratio may also serve as an indicator of the effectiveness of anti-TB treatment (46) since it normalizes following treatment. Studies by La Manna et al. (47) and Wang et al. (48) suggest that the ML ratio can also be used as a marker to predict the risk of developing active TB. A study by Rakotosamimanana et al. (49), suggested that TST along with ML ratio may predict the risk for the development of TB in contacts. *In vitro* studies performed by Naranbhai et al. (50) showed that ML ratio is related to MTB growth and thereby related to the stage of TB. Naranbhai et al. (51) performed a study of ML ratio in 3–4 month infants and found that an elevated ML ratio is directly correlated with the increased risk for the development of TB before 2 years of age. Though ML ratio seems to be a well-suited prognostic marker in defining the risk for TB as well as the efficiency of treatment, the reliability of this marker remains to be confirmed and it is not known whether it correlates with any other diseases or inflammatory conditions. Further prospective studies in larger cohorts will help to define the role of ML ratio in TB prognosis and management.

## Biomarkers

Biomarkers are considered to be a critical component of management of all diseases including TB. Biomarkers are the key factors that can help in determining disease stage, early diagnosis, predicting the behavior of pathogens within the host immune system, and the response of host immune cells toward the pathogen and the disease. Biomarkers may also help to determine treatment and to protect from future complications. Numerous transcriptomic approaches have revealed biomarkers for TB expressed by monocytes. ML ratio as discussed in the previous section can also be considered as a biomarker for TB. Some of the biomarkers identified by the researchers for TB are described in Table 2.

**Table 2:** List of identified biomarkers based on monocytes for tuberculosis (TB).

Biomarkers	Signature	Functions	Expressed by	Able to	Reference
FPR1	Formyl peptide receptor 1	Pro-inflammatory, antimicrobial defense	Professional phagocytes		Jacobsen et al. (52)

Combination of CD64, LTF, and RaB33a	CD64		Monocytes	Discriminate active TB, latent TB infection, and healthy status	
	Fc gamma receptor Ia	Induce phagocytosis, respiratory burst and antibody-dependent cell-mediated cytotoxicity in monocytes, macrophages, and granulocytes		

	LTF			
	Lactoferrin is a transport molecule with high affinity for iron	Modulates the host response by competing with the microbes for iron		

	Rab33a			
	Member of Ras-associated small GTPase family	Regulates intracellular trafficking		

mCD14 receptor	Membrane bound CD14 receptor—55 kD glycoprotein	Pattern recognition receptor for *Mycobacterium* tuberculosis components such as LAM and lipoproteins	Monocytes	Biomarker for active TB	Druszczynska et al. (53)

HMBG1	High mobility group box 1 protein also known as Amphoterin	Pro-inflammatory cytokine, serves as DAMP to alert the innate immune system by recruiting inflammatory cells during disease condition, acts as an immune adjuvant to trigger the response of T cells, dendritic cells, and endothelial cells	Monocytes	Biomarker for active TB	Zeng et al. (54)

IL26	Member of interleukin-10 cytokine family	Inhibitory effect on anti-mycobacterial activity	Monocytes	Susceptible gene for TB	Guerra-Laso et al. (55)

CD163	Monocyte/macrophage specific glycoprotein	Scavenger receptor	Monocytes	Biomarker for TB disease progression and monitor treatment efficacy	Lastrucci et al. (27)

Although many biomarkers for TB based on monocytes have been suggested are available, they need to be validated in larger patient cohorts. In addition, the cost, time, and the conditions for performing reproducible assays need to be established so that they can be used in rural areas where TB is endemic. These are challenging considerations that will require much future work.

## Current Research Gaps and Future Research

Although many studies exploring monocytes and their subsets have been performed in TB, further studies on monocyte subsets, particularly CD16^+^ intermediate monocytes are still needed to fill the gaps in knowledge.

- Limited studies have been performed that investigate the role of CD16^+^ monocyte subsets in TB. Animal studies may be helpful to correlate their function and phenotype with respect to disease progression and protection.
- Recent studies have documented perturbation of the CD16 intermediate monocyte subset upon infection with MTB, suggesting that this cell type favors the intracellular survival of MTB. More studies are needed to document the dysfunction of this subset with reference to their differentiation ability, antigen recognition potency, migration behavior, phagocytic properties, immunostimulation, autophagy, apoptosis, metabolic program, and the mechanism of pathogen clearance. These studies will be helpful to develop newer host directed therapies.
- Although IL-10 contribute to the expansion of CD16^+^ monocytes, other factors such as the antigenic components of MTB (secreted and membrane bound antigens) and the host environment (antigen presentation), can favor the expansion of CD16^+^ monocytes. More work is needed to identify the factors involved in the expansion of CD16^+^ monocytes in established mycobacterial infection.
- Molecular profiling of sorted monocyte subsets of infected patients will be helpful to identify their unique functions. Exploring the phenotype and function of monocyte subsets in granulomas through *in vivo* animal studies could provide the clues about the intracellular survival of MTB and help to decipher the pathophysiology of TB. Newer techniques such as CyTOF and MALDI-TOF could be employed to identify additional phenotypes within sorted monocyte subsets. Single-cell transcriptomic studies involving RNAseq, miRNome, and/or epigenetic profiling needs to be performed in order to understand the functional roles and transcriptional regulation of the different subsets.
- Metabolomic profiles of classical and non-classical monocytes have been very well studied. Substantial insights into the underlying mechanisms of metabolic switches within intermediate monocyte subsets may provide novel tools to identify their cellular function during mycobacterial infection. Drugs inducing metabolic switches can be tested for the treatment of TB.
- Studies of monocyte-related biomarkers in patients of different ethnicities should be encouraged. Previously reported biomarkers need to be validated in larger clinical cohorts including extra pulmonary TB and other infectious or inflammatory conditions in order to delineate their specificity.

## Conclusion

Our review describes the phenotypic and functional variations within monocyte subsets, with reference to mycobacterial infection. With further understanding, we may be able to identify a possible mechanism for the intracellular survival of MTB and to decipher the clues behind the host immune response that influences TB pathogenesis. Current knowledge gaps and suggested future research on monocyte subsets also include better definition of biomarkers for early diagnosis or prognosis of disease and exploration of newer host directed therapies to combat mycobacterial infection.

## Author Contributions

PS and RB: drafting and revising the article, concept and design. KM: contributed to writing. UR: revision of the article; concept and design.

## Conflict of Interest Statement

The authors declare that the research was conducted in the absence of any commercial or financial relationships that could be construed as a potential conflict of interest.
